# Aging affects GABAergic function and calcium homeostasis in the mammalian central clock

**DOI:** 10.3389/fnins.2023.1178457

**Published:** 2023-05-16

**Authors:** Anneke H. O. Olde Engberink, Pablo de Torres Gutiérrez, Anna Chiosso, Ankita Das, Johanna H. Meijer, Stephan Michel

**Affiliations:** Laboratory for Neurophysiology, Department of Cell and Chemical Biology, Leiden University Medical Center, Leiden, Netherlands

**Keywords:** excitatory/inhibitory balance, suprachiasmatic nucleus, calcium imaging, old mice, circadian, chloride transporters

## Abstract

**Introduction:**

Aging impairs the function of the central circadian clock in mammals, the suprachiasmatic nucleus (SCN), leading to a reduction in the output signal. The weaker timing signal from the SCN results in a decline in rhythm strength in many physiological functions, including sleep–wake patterns. Accumulating evidence suggests that the reduced amplitude of the SCN signal is caused by a decreased synchrony among the SCN neurons. The present study was aimed to investigate the hypothesis that the excitation/inhibition (E/I) balance plays a role in synchronization within the network.

**Methods:**

Using calcium (Ca^2+^) imaging, the polarity of Ca^2+^ transients in response to GABA stimulation in SCN slices of old mice (20–24 months) and young controls was studied.

**Results:**

We found that the amount of GABAergic excitation was increased, and that concordantly the E/I balance was higher in SCN slices of old mice when compared to young controls. Moreover, we showed an effect of aging on the baseline intracellular Ca^2+^ concentration, with higher Ca^2+^ levels in SCN neurons of old mice, indicating an alteration in Ca^2+^ homeostasis in the aged SCN. We conclude that the change in GABAergic function, and possibly the Ca^2+^ homeostasis, in SCN neurons may contribute to the altered synchrony within the aged SCN network.

## 1. Introduction

In mammals, the suprachiasmatic nucleus (SCN) functions as a master circadian clock that drives 24 h rhythms in both physiology and behavior. Based on molecular feedback loops, individual SCN neurons generate _~_24 h rhythms in gene expression and cellular processes that in turn regulate electrical activity rhythms (Buhr and Takahashi, 2013; Hastings et al., 2018). This circadian rhythmicity is maintained when the SCN neurons are isolated, demonstrating that single cells function as cell autonomous oscillators (Welsh et al., 1995, 2010; Reppert and Weaver, 2002). Through synchronization and coupling, these individual SCN neurons produce a coherent output signal in ensemble electrical activity with a peak in the subjective day and a trough in the subjective night, which is conveyed to other brain areas and the periphery (Ramkisoensing and Meijer, 2015). Misalignment of single cell oscillators leads to a disruption or loss of SCN rhythm at the tissue level and consequently to malfunction of peripheral clocks. This can have detrimental effects on human health and is associated with, for instance, cancer, cardiovascular, metabolic, and immune disorders (Roenneberg and Merrow, 2016; Patke et al., 2019). Aging promotes such circadian dysfunction by impacting the clock machinery on different levels (Buijink and Michel, 2021). Vice versa, the dysfunctional circadian clock has detrimental effects on the course of aging and is a risk factor for age-related diseases (Kondratova and Kondratov, 2012; Fonseca Costa and Ripperger, 2015). Understanding age-related mechanisms of clock dysfunction can therefore help identifying targets to intervene in this vicious cycle.

In both humans and animal models, age-related changes often lead to a reduction in behavioral activity levels and fragmented sleep–wake rhythms (Dijk and Duffy, 1999; Hofman and Swaab, 2006; Farajnia et al., 2012), a longer latency to re-entrain to shifted light–dark schedules (Biello, 2009; Froy, 2011), and the inability to adapt to a different photoperiod (Buijink et al., 2020). These behavioral deficits are likely the effect of age-related attenuation of the timing signal generated by the SCN network (Oster et al., 2003; Farajnia et al., 2014a).

Both *in vivo* and *ex vivo* studies showed a significant reduction in the amplitude of the ensemble electrical activity rhythm of the aged SCN (Satinoff et al., 1993; Watanabe et al., 1995; Nakamura et al., 2011; Farajnia et al., 2012), which can partly be explained by decreased synchronization within the SCN network. *Ex vivo* electrophysiological recordings of subpopulations in SCN slices of aged mice showed redistribution of phases with a second cluster in the middle of the night. In contrast, peaks in SCN electrical activity in young control slices only clustered around the middle of the day (Farajnia et al., 2012).

An important neurotransmitter that plays a role in synchronization within the SCN network is γ-aminobutyric acid (GABA), which is expressed in almost all SCN neurons (Moore and Speh, 1993; Abrahamson and Moore, 2001). Although the precise role for GABA in the process of synchronization is still under debate (Ono et al., 2020), the number of GABAergic synaptic terminals in the SCN are diminished by 26% due to aging (Palomba et al., 2008) and GABAergic postsynaptic currents are reduced in frequency and amplitude (Nygard et al., 2005; Farajnia et al., 2012). Interestingly, GABA has the ability to act both as an inhibitory and excitatory neurotransmitter in SCN neurons and therefore contributes to plasticity in the excitatory/inhibitory (E/I) balance within the SCN (Albus et al., 2005; Choi et al., 2008). A narrow control over the E/I balance in neuronal networks is known to be critical for proper brain function and E/I imbalance—often caused by a reduction in GABAergic activity—has been correlated with aging-related deficits and the pathogenesis of several neurodegenerative diseases (Rissman and Mobley, 2011; Legon et al., 2016; Tran et al., 2019; Bruining et al., 2020). The possible effects of aging on the polarity of GABAergic signaling and the corresponding E/I balance in the SCN have not yet been studied.

Here, we investigated the aging effect on the GABAergic E/I balance by measuring intracellular calcium levels. Specifically, we determined the polarity of Ca^2+^ transients in response to GABA stimulation in SCN slices of old mice (20–24 months) and young controls during the day, and tested our hypothesis that aging leads to an increment in excitatory responses to GABA. We confirmed that the E/I balance shifted towards more excitation in SCN slices of old mice compared to young controls. Furthermore, we found an effect of aging on the baseline intracellular Ca^2+^ concentration ([Ca^2+^]_i_) and intracellular Ca^2+^. The cells measured from the SCN slices of old mice showed a higher [Ca^2+^]_i_ during the day compared to the cells from the young controls. Analysis of Ca^2+^ transients’ kinetics in response to depolarizations showed slower increase reaching higher peak amplitudes in old SCN neurons, suggesting higher buffer capacity, but also larger Ca^2+^ influx compared to young. This may contribute to high basal [Ca^2+^]_i_ and dysfunctional Ca^2+^ signaling in the old SCN neurons amplifying the effect of increased E/I ratios.

## 2. Materials and methods

### 2.1. Animals

Young (2–4 months, *N* = 11) and old (20–24 months, *N* = 9) male C57BL/6 mice (Janvier Labs, Saint-Berthevin, France) were housed in a climate controlled environment (21°C, 40%–50% humidity) with full-spectrum diffused lighting with an intensity between 50 and 100 lux (Osram truelight TL) and *ad libitum* access to food and water throughout the experiment. The mice were kept in groups of 2–4 mice on an equinoctial photoperiod of 12:12 h light–dark (LD 12:12) cycle. Mice older than 20 months received, in addition to the regular food, hydration and nutritional gels as supportive care. The animals were kept under these conditions for at least 4 weeks prior to the *ex vivo* experiments. *Ex vivo* experiments were performed within a 4-h interval centered around the middle of the day, zeitgeber time (ZT) 6.

### 2.2. Slice preparation

After decapitation, brains were quickly removed and placed into modified ice-cold artificial cerebrospinal fluid (ACSF), containing (in mM): NaCl 116.4, KCl 5.4, NaH_2_PO_4_ 1.0, MgSO_4_ 0.8, CaCl_2_ 1, MgCl_2_ 4, NaHCO_3_ 23.8, glucose 15.1, and 5 mg/L gentamycine (Sigma Aldrich, Munich, Germany) and saturated with 95% O_2_–5% CO_2_. Coronal hypothalamic slices containing the SCN (250 μm) were cut using a vibratome (VT 1000S, Leica Microsystems, Wetzlar, Germany) and sequentially maintained in a holding chamber containing regular, oxygenated ACSF (CaCl_2_ increased to 2 mM and without MgCl_2_). The slices were incubated in a water bath (37°C) for 30 min and were then maintained at room temperature until the start of the recordings.

### 2.3. Ca^2+^ imaging

Neurons in brain slices were bulk-loaded with the ratiometric, membrane permeable Ca^2+^ indicator dye fura-2-acetoxymethyl ester (Fura-2-AM, Teflabs, Austin, United States). First, the slices were transferred from the holding chamber to a 35 mm petri dish and reviewed under the microscope. The side of the slide that contained (the larger part of) the SCN was placed up and the ACSF was removed from the petri dish. One drop of highly concentrated Fura-2-AM (998 μM) was placed on the SCN of each slice for 1 min after which 1 mL of a mix of ACSF containing 7 μM Fura-2-AM was added to the slices. The slices stayed in this mixture for 1 h on room temperature while maintained saturated with 95% O_2_–5% CO_2_. The slices were then rinsed four times with fresh ACSF before being transferred back into the holding chamber where they stayed another hour on room temperature. After this loading protocol, the slices were moved (one by one) to a recording chamber (RC-26G, Warner Instruments, Hamden, CT, United States) mounted on the fixed stage of an upright fluorescence microscope (Axioskop 2-FS Plus, Carl Zeiss Microimaging, Oberkochen, Germany) and constantly perfused with oxygenated ACSF (2.5 mL/min) at room temperature. The indicator dye was excited alternatively at wavelengths of 340 and 380 nm by means of a monochromator (Polychrome V, TILL Photonics; now FEI Munich GmbH, Munich, Germany). Emitted light (505 nm) was collected by a 40x objective and detected by a cooled CCD camera (Sensicam, TILL Photonics; now FEI Munich GmbH, Munich, Germany), and images were acquired at 2 s intervals. It can be expected that Fura-2 will label neurons as well as astrocytes, however the morphology of Fura-2-filled SCN astrocytes is distinct from SCN neurons (Svobodova et al., 2018), and the threshold for Ca^2+^ responses to elevated K^+^ application is higher in astrocytes (Duffy and MacVicar, 1994). We are therefore confident that our recordings were performed on SCN neurons. The slices settled in the recording chamber for at least 5 min before the start of the recordings. After 1 min of baseline recording, GABA (Sigma Aldrich, Munich, Germany;200 μM, 15 s) was applied locally using an eight-channel pressurized focal application system (ALA-VM8, ALA scientific instruments, NY, United States), and Ca^2+^ transients were recorded. After two GABA pulses, 1 min apart, and another minute of recording in which the Ca^2+^ transients returned to baseline, ACSF containing elevated levels of K^+^ (“high K^+^” 20 mM, 15 s) was applied to identify healthy neurons, but also to determine the Ca^2+^ buffer capacity of the cell. Cells with at least 10% increase in [Ca^2+^]_i_ in response to K^+^ were considered to be healthy. The experiments as well as the analysis were accomplished using imaging software (TILLvision, TILL Photonics; now FEI Munich GmbH, Munich, Germany).

### 2.4. Data analysis and statistics

Single-wavelength images were background subtracted and ratio images (340/380) were generated. Regions of interest were generated to define cells and mean ratio values were determined, from which the intracellular Ca^2+^ concentration was calculated. Neuronal Ca^2+^ responses were further analyzed using IGOR Pro (WaveMetrics, Portland, OR, United States). Cells with an amplitude less than 10% of baseline values in response to elevated levels of K^+^, or cells with instable (rising or falling) baselines or baselines >600 nM were excluded from analyses. These criteria will eliminate cells that are severely dysfunction or in apoptosis, but we still expect to be able to record aging-related changes in the SCN slices. Previous work has shown that aging induces rather specific loss of neuronal function (i.e., circadian modulation of ion channel activity) but not overall diminishing of neurons or even GABAergic signaling (Farajnia et al., 2012). The transient responses in Ca^2+^ concentration within the first seconds after the stimulation were evaluated, with responses smaller than ±10% of baseline values defined as non-responding cells. GABA-evoked responses showing Ca^2+^ transients with a decrease in amplitude of more than 10% from baseline were considered inhibitory and responses with an increase of more than 10% from baseline were defined as excitatory. Cells that showed both excitatory and inhibitory responses after a single GABA stimulation were defined as biphasic. Per animal, one to three SCN slices were analyzed and the Ca^2+^ responses to GABA application were measured in typically 60–160 cells. For each animal, the distribution of the different types of responses and the E/I ratio were determined. To calculate the mean E/I ratio, the number of cells that responded excitatory was divided by the number of cells that responded inhibitory per animal (i.e., slices from 1 animal were pooled) and averaged per group. In total we measured 924 cells in 27 slices from 9 old mice and 1,204 cells in 26 slices from 11 young mice.

Ca^2+^ transients in response to high-K^+^ application were analyzed using a custom-made Python script (3.8.10). Basal intracellular Ca^2+^ concentration ([Ca^2+^]_i_) before the GABA application was represented by the mean of 10 measurements before treatment. The amplitude of [Ca^2+^]_i_ transients was defined as the difference between peak and baseline value. Values extending more than three times the standard variation in the data set were excluded. The time constant of the rising phase (tau) was calculated by fitting an exponential curve into the interval between the start of the high-K^+^ rise (defined as the first value 2.5 standard deviations above the baseline) and the maximum value of [Ca^2+^]_i_ for the high-K^+^ transient. Fitted curves with an r-squared value lower than 0.95 were excluded.

Statistical analyses were performed using GraphPad Prism (San Diego, CA, United States) and IBM SPSS statistics version 25 (Armonk, NY, United States). The effect of age on the GABAergic response types, [Ca^2+^]_i_ baselines and high K^+^ responses were tested using generalized estimating equations (GEE) models, with brain slice as a grouping variable. The resulting *p*-values underwent Bonferroni correction to account for multiple testing. When single variable comparisons were drawn between the two age groups, two-sided, unpaired t-tests with Welch’s correction were performed. Differences with *p* ≤ 0.05 were considered significant.

## 3. Results

### 3.1. GABAergic excitation increased in old SCN

**Figure 1:**
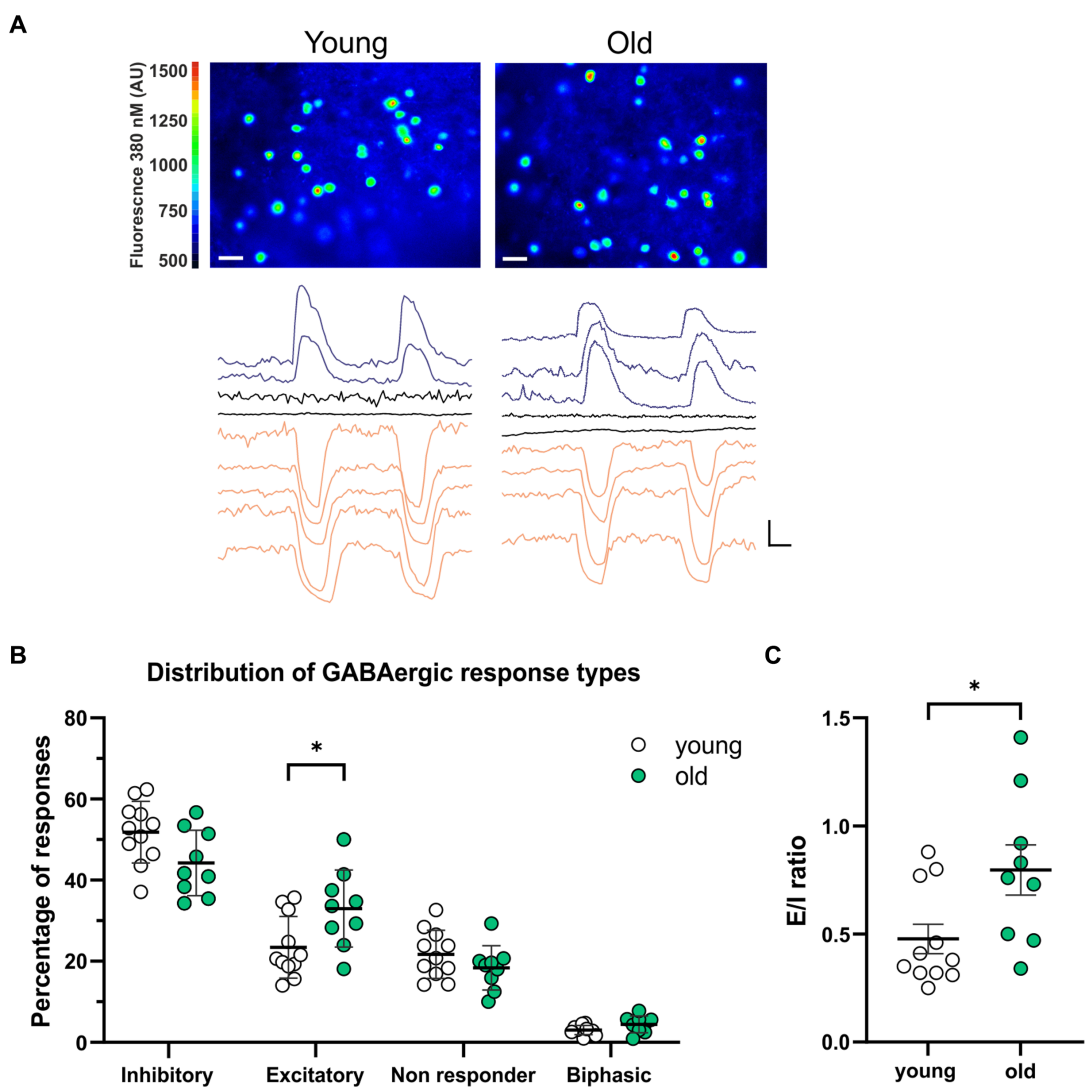
More GABAergic excitation in SCN slices from old mice. **(A)** Upper panels: examples of fura-2-AM loaded SCN neurons in slices from young (left) and old (right) mice. Color scale indicates fluorescence at 380 nm excitation in arbitrary units (Scale bar, 20 μm). Lower panels: example traces of Ca^2+^ transients in response to two GABA administrations recorded from one SCN slice from a young (left) and old (right) mouse. Excitatory responses are shown in blue, inhibitory responses in orange and non-responding cells in black (Scale bars, 50 nM, 20 s). **(B)** The percentages of inhibitory, excitatory, non-responding, and biphasic cells. Each dot represents the mean percentage of responses per response type per SCN. Every single dot in one response type category adds up to 100% together with the corresponding dots in the other categories. **(C)** E/I ratio’s in young and old mice, determined by dividing the number of excitatory responses by the number of inhibitory responses measured from each SCN. Open dots represent values from young mice (*n* = 11) and filled, green dots represent values from old (*n* = 9) mice. Bars indicate mean ± SEM. *p < 0.05, **p < 0.01; distribution of GABAergic response types: GEE with Bonferroni correction **(B)**, E/I ratio: unpaired *t*-test with Welch’s correction **(C)**.

We investigated the effect of aging on the GABAergic activity in SCN neurons by recording GABA-induced single cell Ca^2+^ transients in SCN slices from old (20–24 months) and young (2–4 months) mice (Figure 1A). In each SCN slice, we recorded a combination of transient increases, decreases, or no changes in [Ca^2+^]_i_ in response to GABA application (non-responders). In another subset of SCN neurons we recorded a biphasic response to GABA. The amplitude of the GABAergic responses did not differ between the old and the young SCN neurons (Supplementary Figure S1, inhibition; old: −66.53 ± 5.39 nM, *n* = 433, young: −59.87 ± 1.82 nM, *n* = 691, *p =* 1.0, excitation; old: 58.90 ± 4.32 nM, *n* = 313, young: 62.73 ± 5.03 nM, *n* = 322, *p =* 1.00). The percentages of the different GABAergic response types differed between old and young animals. Old SCN slices exhibited significantly more excitatory responses to GABA as compared to SCN slices of young controls (Figure 1B; Supplementary Figure S2, old: 32.99 ± 3.17%, *n* = 9, young: 23.41 ± 2.29%, *n* = 11, *p* = 0.044). This increase in GABAergic excitation leads to an increase in the E/I balance in old SCN slices, as compared to young SCN slices (Figure 1C, old: 0.80 ± 0.12, *n* = 9, young: 0.48 ± 0.07, *n* = 11, *p =* 0.034). These results show that aging affects the polarity of responses to GABA.

### 3.2. Age-related increase in GABAergic excitation mainly in posterior part of the SCN

Several studies have shown that the amount of GABAergic excitation varies between the different SCN areas in rats (Albus et al., 2005; Choi et al., 2008; Irwin and Allen, 2009). Therefore, we tested whether there were regional differences in the distribution of the GABAergic response types along the anteroposterior and the dorsoventral axis. We defined regions by earlier described landmarks like the shape of the optic chiasm and the distance to the 3rd ventricle (Morin et al., 2006; see Supplementary Figure S3).

**Figure 2:**
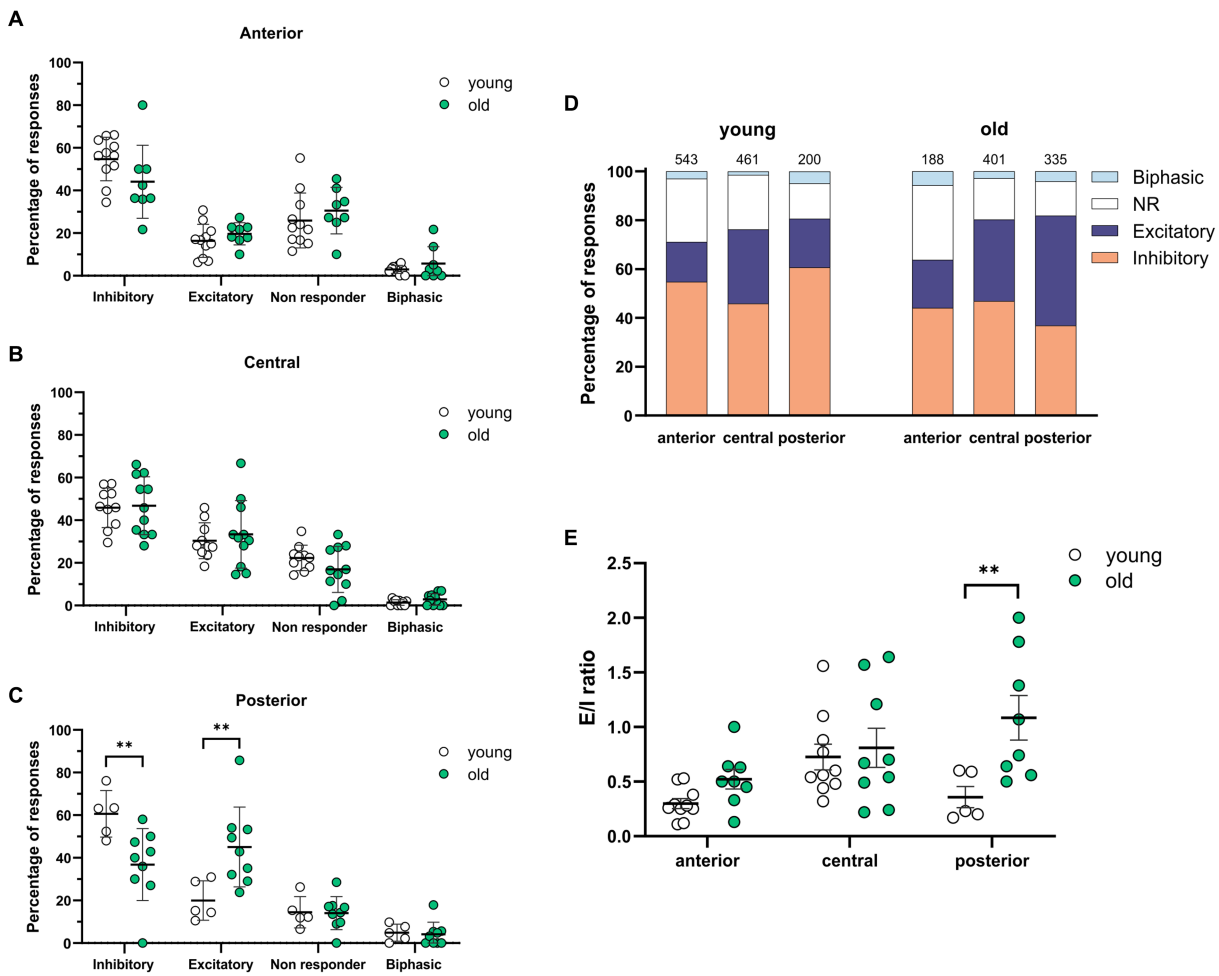
Spatial differences in GABAergic responses along the anteroposterior axis. **(A–C)** The percentages of inhibitory, excitatory, non-responding, and biphasic cells in the anterior **(A)**, central **(B)**, and posterior **(C)** part of the young and old SCN. Each dot represents the mean percentage of responses per response type per SCN. Every single dot in one response type category adds up to 100% together with the corresponding dots in the other categories. **(D)** Distribution of GABAergic response types for the anterior, central, and posterior part of the young (left) and the old (right) SCN. Orange represents the percentage of inhibitory responses, dark blue represents excitatory responses, white represents non-responding cells, and light blue represents biphasic responses. The value on top of the bar shows the total number of cells measured. **(E)** E/I ratios per SCN region in young and old mice, determined by dividing the number of excitatory responses by the number of inhibitory responses measured from different parts of the SCN along the anteroposterior axis. Open dots represent values from young mice and filled, green dots represent values from old mice. Bars indicate mean ± SEM. **p < 0.01; distribution of GABAergic response types: GEE with Bonferroni correction **(A,B,C,E)**.

**Figure 3:**
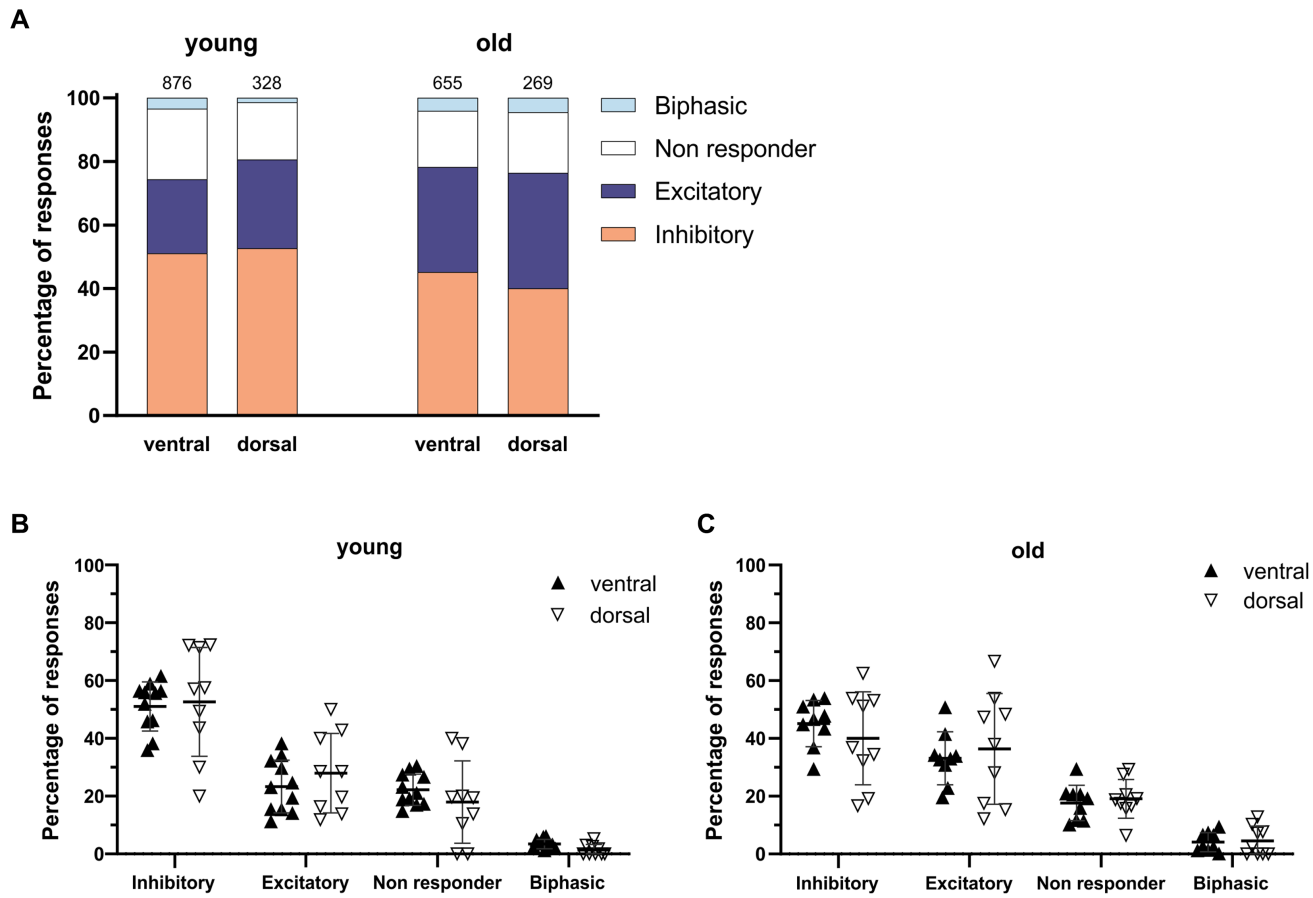
GABAergic responses along the dorsoventral axis. **(A)** Distribution of GABAergic response types for the ventral and dorsal part of the young (left) and the old (right) SCN. Orange represents the percentage of inhibitory responses, dark blue represents excitatory responses, white represents non-responding cells, and light blue represents biphasic responses. The value on top of the bar represents the total number of cells measured. **(B,C)** The percentages of inhibitory, excitatory, non-responding, and biphasic cells in the dorsal and ventral part of the young **(B)** and old **(C)** SCN. Each dot represents the mean percentage of responses per response type per SCN sub-region. Every single dot in one response type category adds up to 100% together with the corresponding dots in the other categories. Filled triangles represent values from the ventral part and open triangles represent values from the dorsal part of the SCN. Bars indicate mean ± SEM. GEE for each category with Bonferroni correction **(B,C)** n. s.

When comparing young and old, the posterior part of the SCN was the only region showing significant differences with more GABAergic excitation and less inhibition (Figures 2C,D, excitation; old: 45.05 ± 6.24%, *N* = 9, young: 19.98 ± 4.14%, *N* = 5, *p* = 0.001, inhibition; old: 36.81 ± 5.53%, *N* = 9, young: 60.62 ± 4.89%, *N* = 5, *p* = 0.04).The calculated E/I balance was also significantly different between young and old in the posterior SCN slices (Figure 2E, old: 1.08 ± 0.20, *N* = 8, young: 0.36 ± 0.10, *N* = 5, *p* = 0.003). No differences in GABAergic responses or E/I balance were observed in the central part of the SCN slices of young and old mice (Figures 2B,E, E/I ratio; old: 0.79 ± 0.19, *N* = 10, young: 0.73 ± 0.12, *N* = 10, *p* = 0.767). There were also no significant differences in GABAergic responses between the dorsal and ventral SCN, both for the old and young mice (Figure 3).

### 3.3. Altered Ca^2+^ homeostasis in old SCN neurons

**Figure 4:**
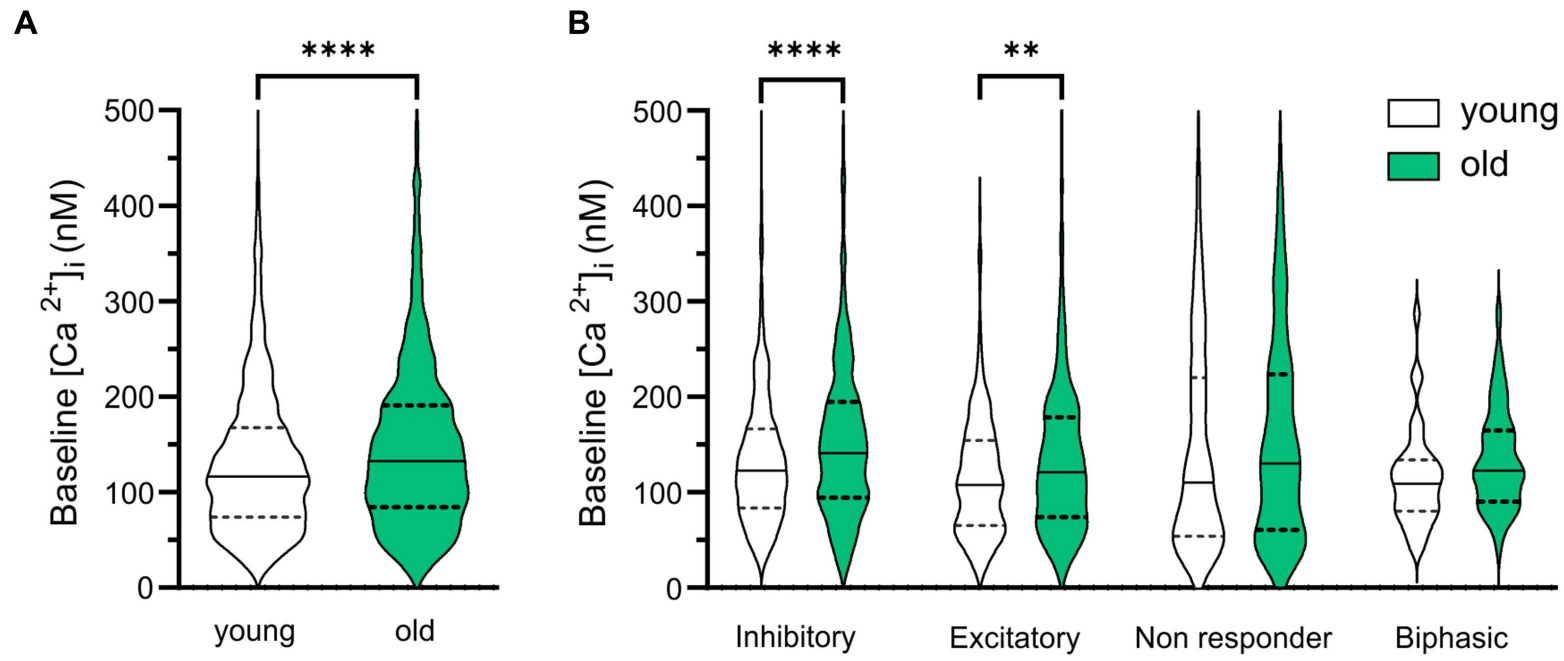
Baseline [Ca^2+^]_i_ is higher in old SCN neurons. **(A)** Violin plots show baseline [Ca^2+^]_i_ levels (nM) from all SCN neurons measured in slices from young (*n* = 1,204) and old (*n* = 924) mice. **(B)** Violin plots show baseline [Ca^2+^]_i_ levels (nM) from all SCN neurons measured categorized per GABAergic response type. White violins represent data from young mice and grey violins represent data from old mice. Violin plots show median and quartiles, **p < 0.01, ****p < 0.0001, unpaired *t*-test with Welch’s correction **(A)**, GEE with Bonferroni correction **(B)**.

To examine whether the baseline [Ca^2+^]_i_ changes with aging, we compared [Ca^2+^]_i_ (determined by the average [Ca^2+^]_i_ in a 20 s interval before GABA application) in SCN neurons from old and young mice during the day. We found higher baseline [Ca^2+^]_i_ levels in cells of the old SCN, when compared to the young SCN neurons (Figure 4A, old: 150.17 ± 3.78 nM, *n* = 924, young: 132.09 ± 2.28 nM, *n* = 1,204, *p* < 0.0001). We also wondered if GABAergic response types were correlated to different baseline levels of [Ca^2+^]_i_. Interestingly, the age-related increase in basal [Ca^2+^]_i_ was restricted to cells responding to GABA with either inhibition or excitation, but not found in non-responding cells or cells with a biphasic response to GABA. Baseline [Ca^2+^]_i_ was higher in old SCN neurons that exhibited GABAergic inhibitory as well as excitatory responses, when compared to young SCN neurons (Figure 4B, inhibition; old: 160.80 ± 6.80 nM, *n* = 419, young: 134.10 ± 2.78 nM, *n* = 632, *p* = 0.000092, excitation; old: 135.10 ± 4.75 nM, *n* = 296, young; 116.10 ± 3.88 nM, *n* = 270, *p* = 0.004).

**Figure 5:**
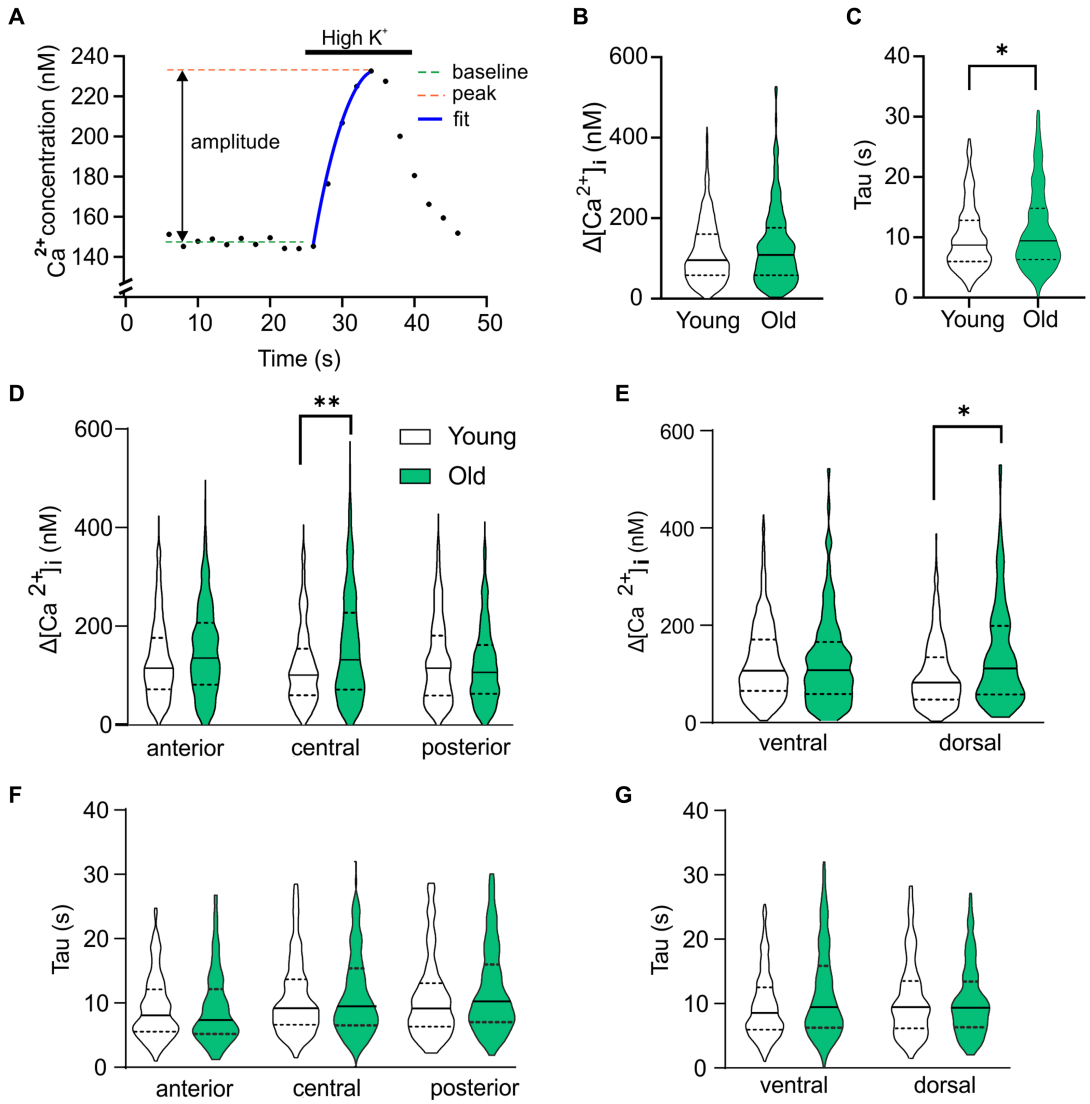
Age-dependent changes in Ca^2+^ homeostasis. **(A)** Example of depolarization-induced Ca^2+^ transient to explain the parameters analyzed and plotted in **(B–G)**. **(B)** Violin plot showing the amplitude of the Ca^2+^ response to depolarization caused by elevated extracellular K^+^ for all cells recorded. **(C)** Rise time of the depolarization-induced Ca^2+^ transient is significantly increased in old SCN neurons compared to young controls. **(D,E)** Analysis of subregions show significant higher amplitude of High K^+^ response in old neurons of posterior and dorsal SCN compared to young controls. **(F,G)** Time constant of rise in [Ca^2+^]_i_ is significantly increased in ventral neurons of SCN neurons from old mice compared to young controls. *p < 0.05, **p < 0.01, independent samples *t*-test **(B,C)**, GEE with Bonferroni correction **(D–G)**.

These results suggest that Ca^2+^ homeostasis in the SCN neurons is affected by age and the GABAergic response type is correlated to the baseline [Ca^2+^]_i_ in young and old SCN. To further investigate this, we analyzed the kinetics and amplitude of the high-K^+^ response, which can be influenced by alteration in the intracellular Ca^2+^ buffering system. We found that the rise time of the Ca^2+^ influx increased in aging SCN neurons (Figure 5C; Young; median = 9.48 s, IQR = 8.92 s. Old; median = 10.24, IQR = 11,06, *p* = 0.045), while there were no differences in amplitude of the peak response (Figure 5B, *p* = 0.205). However, we found regional differences in amplitude of Ca^2+^ transients between young and old SCN with larger impact in the central and dorsal regions on the amplitude of the response (Figures 5D,E, central: *p* = 0.006; dorsal: *p* = 0.018). We could not find a significant change in rise time in the subregions (anterior: *p* = 1.0; central: *p* = 0.609; posterior: *p* = 0.795). This implies an increased buffering capacity of the SCN causing the slower rise, but also an increased net Ca^2+^ influx in the central subregion leading to a larger response during excitation of the neurons.

## 4. Discussion

In this study we examined the effect of aging on the GABAergic E/I balance and on intracellular Ca^2+^ levels in the mammalian pacemaker. Our results demonstrate that aging affects the response polarity, and thus the function, of GABA, which is the most abundant neurotransmitter in the SCN. We measured significantly more GABAergic excitatory responses in SCN slices from old mice when compared to slices from young controls. Particularly, in the posterior part of the old SCN we found significantly more excitation and significantly less inhibition compared to the young SCN. Accordingly, we recorded increased E/I ratios in SCN slices from old mice. We also demonstrate that the baseline [Ca^2+^]_i_ is higher in SCN cells from old mice, compared to young mice. This is interesting considering that Ca^2+^ is an important intracellular signaling molecule and critical for molecular rhythm generation (Lundkvist et al., 2005). Given previous studies on the aging SCN, we are confident that our data reflect the impact on aging on neuronal physiology and we try to exclude pathological phenomena by our exclusion criteria. However, the aging process is a continuum and the border to pathology is not always clear-cut. The number of cells unresponsive to GABA application was not significantly different between young and old animals, suggesting that aging did not severely compromise GABAergic signaling *per se* and our results are reflecting specific aging-dependent changes like reported earlier for circadian modulation of ion channel activity (Farajnia et al., 2012). Still, additional experiments for future studies could include perforated patch recordings for determining E/I (Farajnia et al., 2014b) and Fura-2 titration via a patch electrode to measure the calcium homeostasis in more detail (Neher, 1995).

GABA is known to elicit both inhibitory and excitatory responses in the central clock (Choi et al., 2008; Irwin and Allen, 2009; Farajnia et al., 2014b) and the polarity of GABAergic activity can switch depending on the subregion of the SCN (Albus et al., 2005; Choi et al., 2008; Irwin and Allen, 2009; DeWoskin et al., 2015; Myung et al., 2015). We found spatial differences in the distribution of the different GABAergic response types, with a significant increase in excitatory responses along the anteroposterior axis in the old, but not the young SCN (Figures 2A–D). In slices from both young and old mice, we did not find a difference in the polarity of the GABAergic responses between the ventral and dorsal SCN (Figure 3). The lack of regional differences in GABAergic responses between anterior, central, and posterior SCN slices of young controls, or between dorsal and ventral SCN slices is comparable to a previous study examining the effect of photoperiod on the GABAergic responses in the mouse SCN (Farajnia et al., 2014b). The spatial organization that we observed in C57BL/6 mice differed from the spatial pattern previously observed in rat, in which there are regional differences found along the dorsoventral axis (Albus et al., 2005; Choi et al., 2008; Irwin and Allen, 2009). We have no explanation for this difference, but it adheres to anatomical differences that are also observed between mice and rats with more defined dorsal and ventral subregions in the rat and more interspersed neuropeptide distribution in the mouse SCN (Morin et al., 2006).

The reported increase in GABAergic excitation in the old SCN suggests an increase in [Cl^−^]_i_, since the polarity of the GABAergic signal depends in part on the [Cl^−^]_i_ and the relationship between the chloride equilibrium potential (E_Cl_^−^) and the membrane potential (V_m_) (Kaila, 1994; Ben-Ari, 2002). Protein expression of the cation-chloride co-transporter responsible for the influx of chloride, the NKCC1, displays circadian rhythmicity in hamsters under constant conditions and is regulated by environmental lighting conditions, as NKCC1 protein levels in the SCN of hamsters housed in constant light are higher than of hamsters entrained to 14:10 LD cycles or under constant darkness (McNeill et al., 2020). There is no clear consensus on the distribution of co-transporter expression in the SCN. NKCC1 protein expression is shown to be higher in the dorsal part of the rat SCN at night, with no differences in subregion during the day (Choi et al., 2008), however, in hamsters exposed to constant darkness or 14:10 LD cycles, NKCC1 expression is higher in the ventral SCN compared to the dorsal part (McNeill et al., 2020). Another study shows higher [Cl^−^]_i_ during the day than during the night in both the ventral and dorsal mouse SCN neurons. Additionally, the KCCs—the extruders of chloride—play a major role in [Cl^−^]_i_ regulation, while NKCC1 has a relatively minor role (Klett and Allen, 2017). Our results suggest that the—relative or absolute—expression of NKCC1 and/or KCC2 may also be affected by aging, since we demonstrated an alteration in the polarity of the GABAergic response in the old SCN.

### 4.1. E/I balance and synchronization within the SCN network

The increase in excitatory responses in the old SCN may be part of mechanisms that contribute to the degree of synchronization within the SCN network. Both *in vivo* and *ex vivo* studies showed a significant reduction in the amplitude of the ensemble electrical activity rhythms of the aged SCN that is likely the consequence of decreased synchronization within the SCN network (Satinoff et al., 1993; Watanabe et al., 1995; Nakamura et al., 2011; Farajnia et al., 2012). *Ex vivo* electrophysiological recordings showed changes in neuronal phase distribution in SCN slices of aged mice (Farajnia et al., 2012) and these alterations at the network level underlie the diminished SCN output signal. Although the mechanisms that regulate neuronal phase distribution in the SCN are still unknown, there are studies suggesting that an increase in E/I balance might be responsible for modulating the phase distribution (Farajnia et al., 2014b; Rohr et al., 2019). GABA is expressed in almost all SCN neurons and is, because of its dual action as inhibitor and activator, an important contributor to the E/I balance in the SCN (Albus et al., 2005; Choi et al., 2008). Even though evidence suggest that GABA is involved in phase adjustment and synchronization of the SCN network, no consensus exists on the precise role of GABA in network synchronization (Ono et al., 2020). Our results contribute to this debate by showing increased levels of GABAergic excitation in an SCN network that is in a more desynchronized state (Farajnia et al., 2012), suggesting GABA regulation is involved in phase distribution. Other studies also suggest that GABA does not promote synchrony, or works as a destabilizer or phase desynchronizer within the SCN, but these studies do not distinguish between GABAergic inhibition or excitation (Aton et al., 2006; Freeman et al., 2013). Whether there is an actual causal link between either the E/I balance and synchronization still needs further investigation.

Alterations in the SCN network are involved in aging, as well as in the adaptation to photoperiods. As with aging, exposure to a long day photoperiod causes more phase dispersal in the SCN (VanderLeest et al., 2007; Brown and Piggins, 2009; Buijink et al., 2016) and a switch in the polarity of GABAergic activity from inhibition to excitation in many SCN neurons (Farajnia et al., 2014b). Farajnia et al. proposes that the relation between GABAergic inhibition and excitation may contribute to the photoperiod-induced phase adjustments within the network. Our results show an increase in the number of excitatory GABAergic responses and an increase in the E/I balance in the old SCN (Figure 1), similar to the changes in the SCN of mice entrained to a long photoperiod, and thus our data support this hypothesis.

### 4.2. Plasticity in E/I balance in the SCN

The polarity of the GABAergic response in the SCN, and thus the E/I balance, can vary depending on time of day or the photoperiod to which the animals are exposed (Albus et al., 2005; Choi et al., 2008; Farajnia et al., 2014b). In addition, a recent study showed that lighting conditions affect the circadian regulation and levels of NKCC1 protein expression, and thus the action of GABA (McNeill et al., 2020). The plasticity in GABAergic function is thought to support adaptation to environmental conditions. It is not clear whether the excitatory action of GABA in aging is also functional by contributing to a compensatory mechanism to reorganize the neuronal network of the SCN, or else, is a consequence of a loss of function in the aging SCN. Moreover, it remains to be investigated whether there is still plasticity in the E/I balance in the SCN of old mice, or if the increase that we show here is static and irreversibly changed in the SCN network. In an recent study we were able to show that the aging SCN is still able to adapt its molecular clock to different photoperiods as well as the young, suggesting that the SCN network is still flexible in aging (Buijink et al., 2020).

### 4.3. Aging and intracellular Ca^2+^ homeostasis in the SCN

One important intracellular component involved in phase adjustment is Ca^2+^, which we determined in our baseline measurements before GABA application. Because of the use of a ratiometric dye, we were able to compare baseline [Ca^2+^]_i_ of SCN neurons from slices of old and young mice. Our results show that the baseline [Ca^2+^]_i_ is higher in SCN cells from old mice, compared to young controls (Figure 4) which is in accordance with previous studies in other brain areas (Galla et al., 2020; Uryash et al., 2020; Mozolewski et al., 2021). In both young and old SCN slices, the neurons that showed GABAergic inhibition exhibited the highest baseline calcium levels. GABA induced Ca^2+^ transients can depend on baseline [Ca^2+^]_i_ (Irwin and Allen, 2009). A possible cause for the relationship between baseline [Ca^2+^]_i_ and GABAergic response type could be the different levels of electrical activity of the neuron. At a higher firing rate, the [Ca^2+^]_i_ baseline would be increased due to influx of Ca^2+^ through voltage-activated Ca^2+^ channels and an inhibitory input would have a larger effect compared to a silent neuron. Also, it is plausible that a neuron with a low or high [Ca^2+^]_i_ may not be able to further lower or raise [Ca^2+^]_i_ after a GABAergic stimulus, respectively.

In addition, we measured changes in kinetics of depolarization induced Ca^2+^ transients. Old neurons in the SCN showed a slower rise in [Ca^2+^]_i_, but also can reach a higher response peak (Figure 5). Similar results were found using patch clamp recordings in old hippocampal neurons in rats (Oh et al., 2013), suggesting an increase in buffer capacity in aged neurons, but also a larger influx of Ca^2+^ exhausting the homeostatic capacity in longer excitations and driving [Ca^2+^]_i_ to high levels.

Our evidence implies that both baseline calcium levels and calcium homeostasis are altered in the old SCN, which could further impair cellular phase adjustments. Even though Ca^2+^ is one of the most essential and well-studied signaling molecules, surprisingly little is known about the influence of aging on the Ca^2+^ signaling or Ca^2+^ homeostasis in the SCN. Studies in other brain areas have focused on the contribution of plasma membrane Ca^2+^ pumps, intracellular stores like the endoplasmic reticulum, and the mitochondria in aged neurons or neurodegenerative disorders (Supnet and Bezprozvanny, 2010; Zaidi et al., 2018; Calvo-Rodriguez et al., 2020; Trombetta-Lima et al., 2021). It should be noted though, that the SCN neurons can already tolerate higher levels of [Ca^2+^]_i_ when compared to other brain areas (Diekman et al., 2013). Given the essential role of calcium in both intracellular signaling pathways and rhythm generation and its association with multiple neurodegenerative disorders (Foster, 2007; Berridge, 2013), restoring calcium signaling in old SCN neurons could be an interesting target for therapy.

Additionally, maintenance of an adequate balance of excitation and inhibition could benefit healthy aging. Several studies have shown a shift in E/I balance, with heightened neuronal activity in the hippocampus or prefrontal cortex due to decreased inhibitory networks (Legon et al., 2016; Tran et al., 2019). This loss of inhibition, and thus an increased E/I ratio, was correlated to aging and the pathogenesis of neurodegenerative disorders (Rissman and Mobley, 2011; Bruining et al., 2020). Moreover, the E/I ratio increased in aged rats with impaired memory function, however aged rats with unimpaired memory function had similar hippocampal E/I ratios as young controls, showing that a proper balance between inhibition and excitation is crucial for maintaining memory performance during aging (Tran et al., 2019) and stresses the importance of an adequate balance in the aged brain. Our study demonstrates that the E/I balance of the neuronal network of the central clock is also challenged by aging with potential consequences for clock function. The stabilization of SCN E/I ratio to a healthy range in aging will not only benefit SCN network properties, but may also counteract the detrimental effects of the clock on neurodegenerative diseases (Leng et al., 2019; Fifel and De Boer, 2021).

## Data availability statement

The raw data supporting the conclusions of this article will be made available by the authors, without undue reservation.

## Ethics statement

All animal experiments were performed in accordance with the regulations of the Dutch law on animal welfare, and the institutional ethics committee for animal procedures of the Leiden University Medical Center (Leiden, Netherlands) approved the protocol (AVD 1160020185524; PE. 18.113.07).

## Author contributions

AHOE, SM, and JHM designed the study. AHOE, PdTG, and AC performed the experiments. PdTG developed analysis tools for calcium transients. AHOE, PdTG, AC, and AD performed the analysis of the data. AHOE, SM, JHM, and PdTG wrote the manuscript. All authors contributed to the article and approved the submitted version.

## Funding

This study was supported by funding from Velux Stiftung (project grant 1029 to SM) and by funding from ERC (adv grant 834513 to JHM). This study is part of the doctoral thesis by AHOE (Olde Engberink, 2022).

## Conflict of interest

The authors declare that the research was conducted in the absence of any commercial or financial relationships that could be construed as a potential conflict of interest.

## Publisher’s note

All claims expressed in this article are solely those of the authors and do not necessarily represent those of their affiliated organizations, or those of the publisher, the editors and the reviewers. Any product that may be evaluated in this article, or claim that may be made by its manufacturer, is not guaranteed or endorsed by the publisher.
