# Supplementary material for: Aging affects GABAergic function and calcium homeostasis in the mammalian central clock
Source: Front Neurosci. 2023 May 16;17:1178457. doi: 10.3389/fnins.2023.1178457 (PMC10229097; doi:10.3389/fnins.2023.1178457)
Supplement: Supplementary file 1 [file Data_Sheet_1.pdf]

SUPPLEMENTAL MATERIAL

Aging affects GABAergic function and calcium homeostasis in the mammalian central clock

Anneke H.O. Olde Engberink[†], Pablo de Torres Gutiérrez[†], Anna Chiosso, Ankita Das, Johanna H. Meijer, Stephan Michel*

Department of Cellular and Chemical Biology, Laboratory for Neurophysiology, Leiden University Medical Center, Einthovenweg 20, 2333 ZC, Leiden, the Netherlands.

† These authors contributed equally to this work and share first authorship.

* Corresponding author: S.H.Michel@lumc.nl

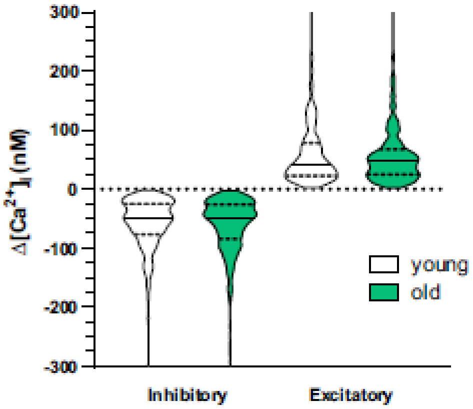

Figure S1: Amplitudes of GABAergic responses. A. Violin plots show amplitudes (delta $[Ca^{2+}]_i$) of all SCN neurons that responded in an inhibitory and excitatory manner.

White violins represent data from SCN slices from young mice and green violins represent data from SCN slices from old mice. GEE with Bonferroni correction., n.s.

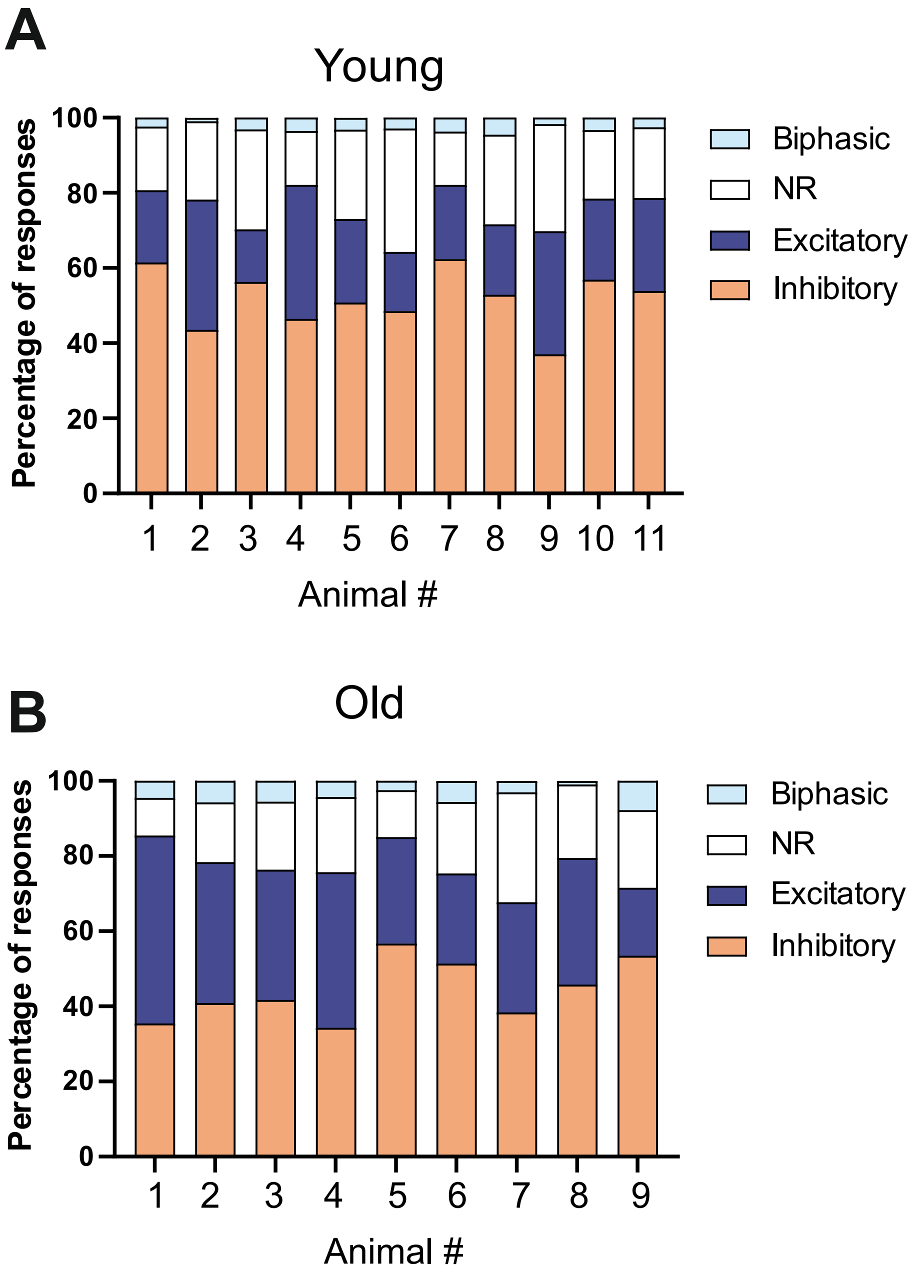

Figure S2: Distribution of Ca^{2+}response types to GABA application in SCN of Young (A) and old (B) mice.

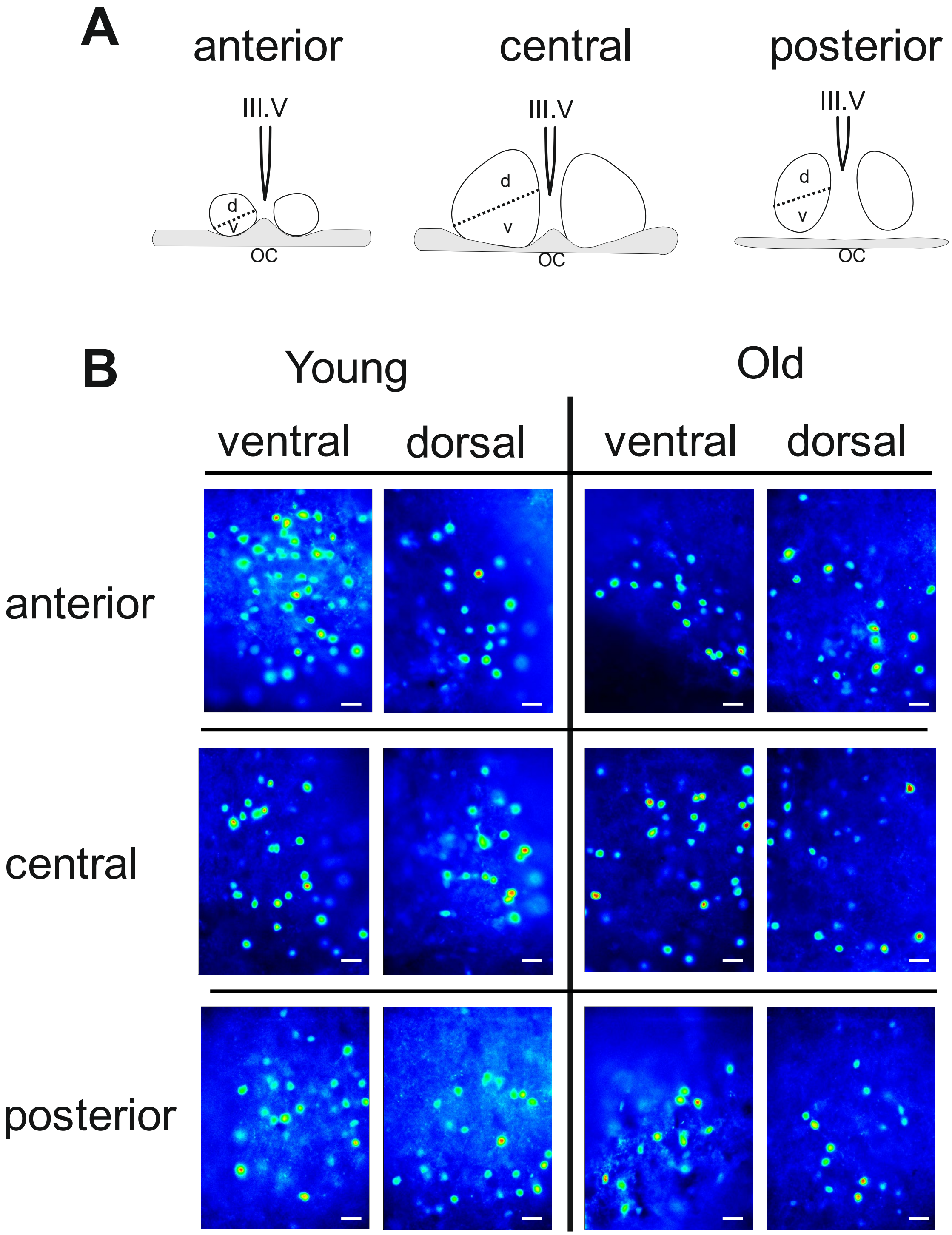

Figure S3: Ca^{2+} imaging of SCN regions. A. Schematic drawings indicating the regions used for recording GABA responses in the SCN. B. Examples of Fura-2 labelled cells in young and old SCN from different regions imaged using a 40x objective . (scale bar = 20 μm)
